# Metabolic reprogramming-related drug resistance in osteosarcoma: from molecular mechanisms to therapeutic translation

**DOI:** 10.3389/fmolb.2026.1928880

**Published:** 2026-09-01

**Authors:** Qun Wu, Hongxiang Chen, Jian Li

**Affiliations:** First Ward of Orthopedics, Loudi Central Hospital, Loudi, Hunan, China

**Keywords:** drug resistance, glutamine metabolism, glycolysis, lipid metabolism, metabolic reprogramming, osteosarcoma, tumor microenvironment

## Abstract

**Background:**

Osteosarcoma remains the most prevalent primary malignant bone tumor in adolescents and young adults. For patients with localized disease, 5-year overall survival reaches 60%–70%; however, for those with metastatic or recurrent disease, 5-year survival remains stagnant at approximately 20%, and chemoresistance represents the primary obstacle to improved outcomes.

**Objective:**

This review systematically elucidates the crosstalk among glycolytic, lipid, and glutamine pathways and their synergistic interactions with the immune microenvironment in driving osteosarcoma drug resistance, while evaluating the translational potential of metabolic targeted therapies.

**Results:**

We highlight that aerobic glycolysis-derived histone lactylation, which has been shown to activate multidrug resistance gene transcription in other cancers and represents a candidate epigenetic mechanism warranting investigation in osteosarcoma; SCD1-mediated monounsaturated fatty acid synthesis confers ferroptosis resistance; glutamine-derived α-KG supports epigenetic programming and redox homeostasis; and metabolic competition creates an immunosuppressive tumor microenvironment. We further discuss metabolic heterogeneity, plasticity, and metabolomic methodologies as applied to osteosarcoma.

**Conclusion:**

Single-agent metabolic inhibitors show limited clinical efficacy due to metabolic plasticity and compensatory activation. Almost all metabolic targeting evidence remains preclinical; no metabolic therapy has yet entered standard osteosarcoma care. Triple combination strategies (metabolic inhibitors + immunotherapy + chemotherapy) represent a mechanistically attractive but clinically untested hypothesis. Telaglenastat (CB-839), a glutaminase inhibitor, has been prioritized for osteosarcoma clinical trials but efficacy data in osteosarcoma patients remain pending.

## 1 Introduction

Osteosarcoma is the most common primary malignant bone tumor, accounting for approximately 35%–40% of all primary malignant bone tumors (Siegel et al., 2022), with a peak incidence at 10–25 years of age and a male-to-female ratio of approximately 1.5:1. The age-standardized incidence is approximately 3.4 per million person-years (Mirabello et al., 2009). Each year, there are roughly 800–1,000 new cases in the United States (Gianferante et al., 2020) and an estimated 4,000–5,000 new cases in China based on national cancer registry data (Lilienthal and Herold, 2020).

Over the past decade, metabolic reprogramming has been recognized as a core hallmark of cancer, representing a key adaptive strategy for tumor cells to meet the biosynthetic, bioenergetic, and redox demands of rapid proliferation under stressful microenvironmental conditions including hypoxia, nutrient deprivation, and chemotherapy exposure. Unlike normal cells which primarily rely on mitochondrial oxidative phosphorylation for energy production under aerobic conditions, osteosarcoma cells undergo profound metabolic rewiring characterized by the Warburg effect (aerobic glycolysis), enhanced *de novo* lipogenesis, and glutamine addiction. These metabolic alterations not only provide ATP and building blocks for macromolecule synthesis but also directly contribute to drug resistance through multiple mechanisms: maintenance of redox homeostasis, regulation of epigenetic modifications, acidification of the tumor microenvironment, and suppression of anti-tumor immunity (Pavlova and Thompson, 2016; Feng et al., 2022; Yin et al., 2024).

**FIGURE 1:**
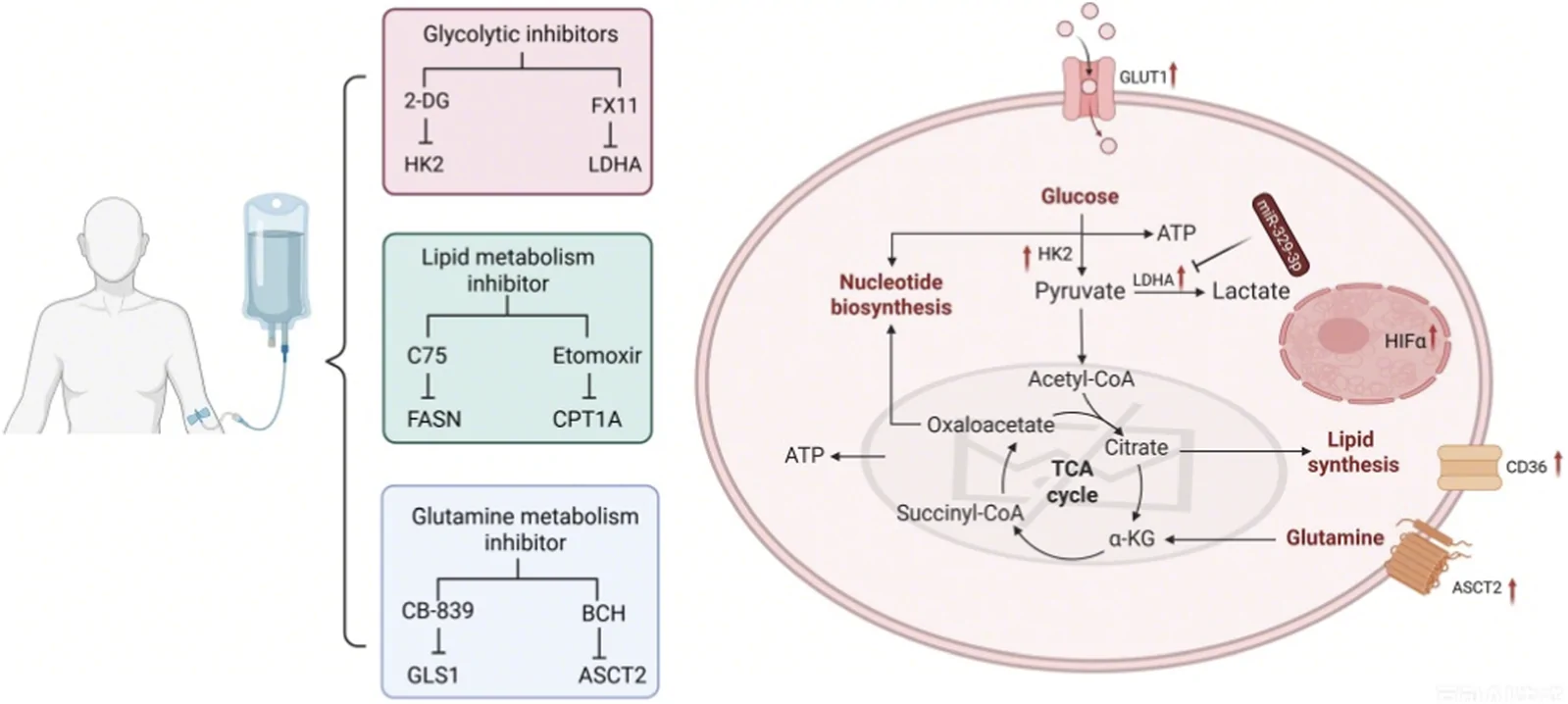
Overview of metabolic reprogramming and drug resistance in osteosarcoma. Three core metabolic pathways—glycolysis, lipid metabolism, and glutaminolysis—are coordinately upregulated through PI3K/AKT/mTOR, HIF-1α, and c-Myc signaling hubs. These pathways drive chemoresistance through multiple mechanisms: enhanced biosynthesis and energy supply, antioxidant defense, epigenetic regulation (histone lactylation, α-KG-dependent demethylation), and creation of an immunosuppressive tumor microenvironment. Metabolic plasticity and compensatory pathway activation represent major barriers to single-agent metabolic therapy.

While previous reviews have individually described glycolytic, lipid, or glutamine metabolic alterations in osteosarcoma, the present review systematically integrates all three major metabolic pathways and their crosstalk in the context of drug resistance, with a focus on epigenetic mechanisms, metabolic plasticity, and translational challenges (Figure 1).

### 1.1 Metabolic heterogeneity in osteosarcoma

Osteosarcoma is not a metabolically uniform disease. Significant metabolic heterogeneity exists along multiple axes, with important implications for both resistance mechanisms and therapeutic targeting.

#### 1.1.1 Pediatric versus adult osteosarcoma

Pediatric osteosarcoma, the more common presentation, is characterized by high proliferative drive and robust mTOR pathway activation. Immunohistochemical profiling of pediatric OS tumors demonstrates concurrent upregulation of glycolytic, glutaminolytic, and lipogenic enzymes downstream of mTOR (Mohás et al., 2022). Adult-onset osteosarcoma, often secondary to Paget’s disease or prior radiation, may exhibit a distinct metabolic profile—potentially more oxidative and less glycolytic—though systematic comparative metabolomic studies are lacking.

#### 1.1.2 Primary versus metastatic lesions

Metastatic osteosarcoma, most commonly seeding the lungs, undergoes metabolic adaptation to the target organ microenvironment. Lung metastases may shift toward increased oxidative phosphorylation (OXPHOS) and fatty acid oxidation to exploit the high-oxygen, lipid-rich pulmonary environment—a phenomenon termed metabolic organotropism. Direct comparative metabolomic data between paired primary and metastatic OS samples remain scarce.

#### 1.1.3 Treatment-naïve versus chemoresistant tumors

Acquired chemoresistance drives additional metabolic rewriting beyond baseline oncogenic reprogramming. Resistant clones typically exhibit enhanced metabolic flexibility, upregulated antioxidant defense systems, and increased reliance on alternative nutrient sources. Longitudinal metabolic evolution during chemotherapy is not captured by single-timepoint diagnostic biopsies.

#### 1.1.4 Genomic and molecular subtypes

TP53-mutant tumors show enhanced glycolysis and nucleotide biosynthesis. MYC-amplified subsets exhibit heightened glutamine dependency, potentially identifying candidates for glutaminase inhibitor therapy. RB1 loss is associated with altered mitochondrial metabolism. However, the highly chaotic genomic landscape of OS makes clean metabolic subtype stratification challenging.

#### 1.1.5 Intratumoral metabolic heterogeneity

Perivascular tumor regions are more oxidative and glutamine-dependent, while hypoxic zones rely heavily on glycolysis. Cancer stem cell subpopulations may possess unique metabolic dependencies, including enhanced fatty acid oxidation. This intratumoral diversity means that single-agent metabolic therapies will likely select for pre-existing resistant subclones.

### 1.2 Metabolomic technologies and methodologies

The study of metabolic reprogramming in osteosarcoma relies on a suite of metabolomic technologies, each with distinct strengths, applications, and limitations. Understanding these methodologies is essential for interpreting the evidence base.

#### 1.2.1 Untargeted metabolomics (GC-MS and LC-MS)

Global metabolite profiling has been applied to OS cell lines, patient serum, and tumor tissue, identifying elevated glycolytic intermediates, altered TCA cycle metabolites, and dysregulated amino acid profiles. Limitations include batch effects, non-standardized normalization, and incomplete metabolite annotation.

#### 1.2.2 Stable isotope-resolved metabolomics

Steady-state metabolomics provides only a snapshot of metabolite pool sizes. ^13^C-labeled glucose, glutamine, or palmitate tracing reveals actual pathway flux and carbon fate. In OS, isotope tracing has confirmed the Warburg effect and quantified glutamine’s contribution to TCA cycle anaplerosis.

#### 1.2.3 Imaging metabolomics

^18^F-FDG PET is the clinical standard for assessing glucose metabolism but is non-specific and complicated by bone artifact in primary bone lesions. Emerging tracers include ^18^F-labeled glutamine analogs and hyperpolarized ^13^C-pyruvate, which remain investigational in osteosarcoma.

Spatial and single-cell metabolomics. Emerging technologies such as MALDI-MSI and SIMS enable spatial mapping of metabolites within tumor tissue. These approaches are largely preclinical and have not yet been systematically applied to osteosarcoma.

#### 1.2.4 Multi-omics integration

Metabolomic data integrated with transcriptomic, proteomic, and epigenomic datasets have identified key regulatory nodes (mTOR, HIF-1α, c-Myc) coordinating metabolic pathway rewiring. However, integrative analyses in OS are constrained by limited sample sizes.

## 2 Glycolytic metabolic reprogramming and drug resistance in osteosarcoma

**FIGURE 2:**
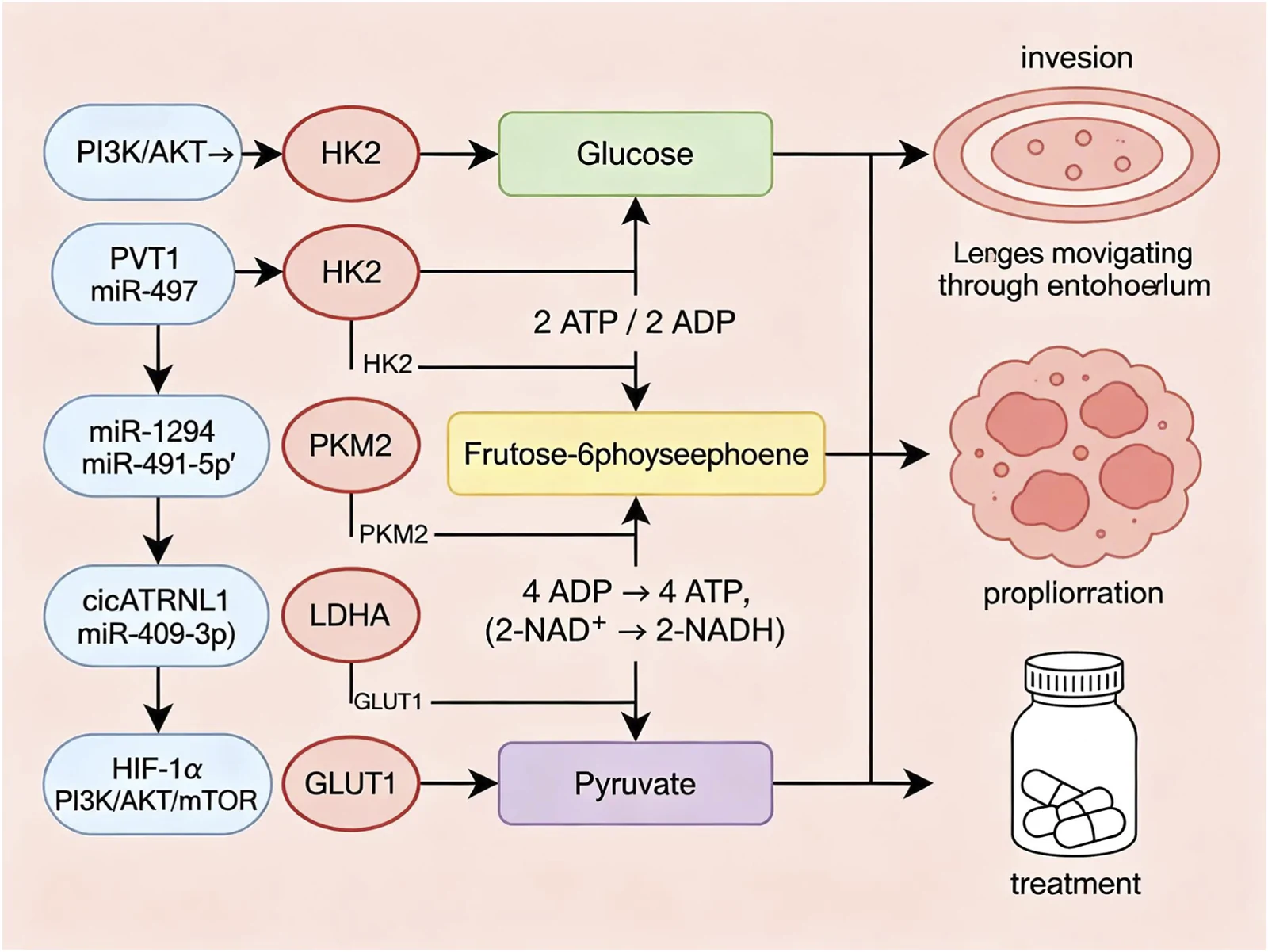
Glycolytic metabolic reprogramming and regulatory mechanisms in osteosarcoma. Key glycolytic enzymes (HK2, PKM2, LDHA, GLUT1) are upregulated at multiple levels: signaling pathways (PI3K/AKT, HIF-1α/mTOR) and non-coding RNAs (miR-497, miR-1294, miR-491–5p, miR-409–3p, circATRNL1). Enhanced glycolysis promotes tumor proliferation, invasion, and chemoresistance. Glucose is metabolized to fructose-6-phosphate (investment phase, consuming 2 ATP), and subsequently to pyruvate (payoff phase, generating 4 ATP and 2 NADH).

Aerobic glycolysis, termed the Warburg effect, describes the phenomenon where tumor cells preferentially metabolize glucose to lactate even in the presence of oxygen and functional mitochondria. This seemingly inefficient metabolic phenotype provides critical advantages for rapidly proliferating tumor cells: rapid ATP production, generation of biosynthetic precursors for nucleotides, amino acids, and lipids, and maintenance of redox balance. In osteosarcoma, glycolytic reprogramming is consistently observed across patient samples and preclinical models, and its degree correlates with chemotherapy response and patient survival (Bishayee et al., 2023) (Figure 2).

### 2.1 Key glycolytic enzymes as drivers of drug resistance

Hexokinase 2 (HK2), the rate-limiting enzyme catalyzing the first step of glycolysis, is significantly upregulated in osteosarcoma tissues compared to adjacent normal bone, with high expression correlating with advanced Enneking stage and poor overall survival. HK2 phosphorylates glucose to glucose-6-phosphate, trapping glucose inside the cell and committing it to glycolytic metabolism. Beyond its canonical enzymatic function, mitochondrial-bound HK2 inhibits apoptosis by interacting with the voltage-dependent anion channel (VDAC), preventing the release of cytochrome c and conferring resistance to chemotherapy-induced apoptosis. The PI3K/AKT/mTOR pathway directly phosphorylates and activates HK2, while non-coding RNAs including lncRNA PVT1 and circRNA_0000285 upregulate HK2 expression by sponging miR-497 and miR-143 respectively (Ni et al., 2024; Zhou et al., 2023). Genetic or pharmacological inhibition of HK2 using 2-deoxyglucose (2-DG) or lonidamine significantly sensitizes osteosarcoma cells to cisplatin and doxorubicin *in vitro* and *in vivo*.

Pyruvate kinase M2 (PKM2) is the embryonic M2 isoform of pyruvate kinase that is re-expressed in cancer cells, catalyzing the conversion of phosphoenolpyruvate to pyruvate (Lee et al., 2022). Unlike the constitutively active M1 isoform, PKM2 can exist in a low-activity dimeric state that promotes the accumulation of glycolytic intermediates for biosynthesis. PKM2 is significantly upregulated in osteosarcoma and associated with poor prognosis and chemoresistance. Beyond its cytoplasmic glycolytic function, nuclear PKM2 has been shown to act as a protein kinase phosphorylating histone H3 at threonine 11 (H3-T11) in hepatocellular carcinoma and other cancers, driving MDR1 transcription (Yang et al., 2024). Whether nuclear PKM2 specifically phosphorylates H3-T11 to drive MDR1 expression in osteosarcoma has not yet been directly demonstrated and represents an important area for future investigation.

Lactate dehydrogenase A (LDHA) catalyzes the final step of glycolysis, converting pyruvate to lactate with concomitant oxidation of NADH to NAD+. LDHA is consistently overexpressed in osteosarcoma, and elevated lactate production is a well-documented feature of chemoresistant OS cells. Histone lysine lactylation (Kla) is a recently discovered post-translational modification stimulated by high glycolytic flux (Woodford et al., 2021). In several cancer types and immune cells, lactate-driven histone lactylation directly activates transcription of target genes including MDR1 and ABCG2. In osteosarcoma, while LDHA overexpression and lactate accumulation are firmly established and correlate with drug resistance, direct evidence that histone lactylation specifically activates MDR1/ABCG2 transcription in OS cells is currently lacking. This mechanism should be considered a working hypothesis rather than a demonstrated fact.

### 2.2 Signaling pathways and non-coding RNA regulation

The PI3K/AKT/mTOR signaling axis represents the master regulator of glycolytic reprogramming in osteosarcoma. Activated by receptor tyrosine kinases including IGF-1R and EGFR, this pathway upregulates GLUT1 membrane translocation, activates HK2 and PFKFB3, and promotes c-Myc-dependent transcription of glycolytic enzymes. mTORC1 activation also drives the expression of hypoxia-inducible factor 1α (HIF-1α) even under normoxic conditions, creating a feedforward loop that sustains glycolytic metabolism. HIF-1α is stabilized in the hypoxic bone microenvironment and directly transactivates virtually all glycolytic genes including GLUT1, HK2, PKM2, LDHA, and monocarboxylate transporters MCT1/4. In addition to regulating metabolism, HIF-1α induces the expression of vascular endothelial growth factor (VEGF) and lysyl oxidase (LOX) to promote angiogenesis and metastasis (Peng et al., 2021). The Wnt/β-catenin pathway also contributes to glycolytic regulation, with nuclear β-catenin cooperating with c-Myc to activate glycolytic gene expression.

Non-coding RNAs form a complex regulatory network that fine-tunes glycolytic flux at the post-transcriptional level. Tumor suppressor miRNAs including miR-185, miR-328-3p, and miR-329-3p directly target the 3′-UTRs of HK2, GLUT1, and LDHA respectively, inhibiting their expression and enhancing chemotherapy sensitivity. Conversely, oncogenic non-coding RNAs such as lncRNA CCAT1 and circPVT1 act as competing endogenous RNAs (ceRNAs) to sponge these inhibitory miRNAs, thereby de-repressing glycolytic enzyme expression. For example, circATRNL1 is upregulated in doxorubicin-resistant osteosarcoma cells and sponges miR-409-3p to upregulate LDHA, increasing lactate production and histone lactylation levels to induce drug resistance.

## 3 Lipid metabolic reprogramming and drug resistance in osteosarcoma

**FIGURE 3:**
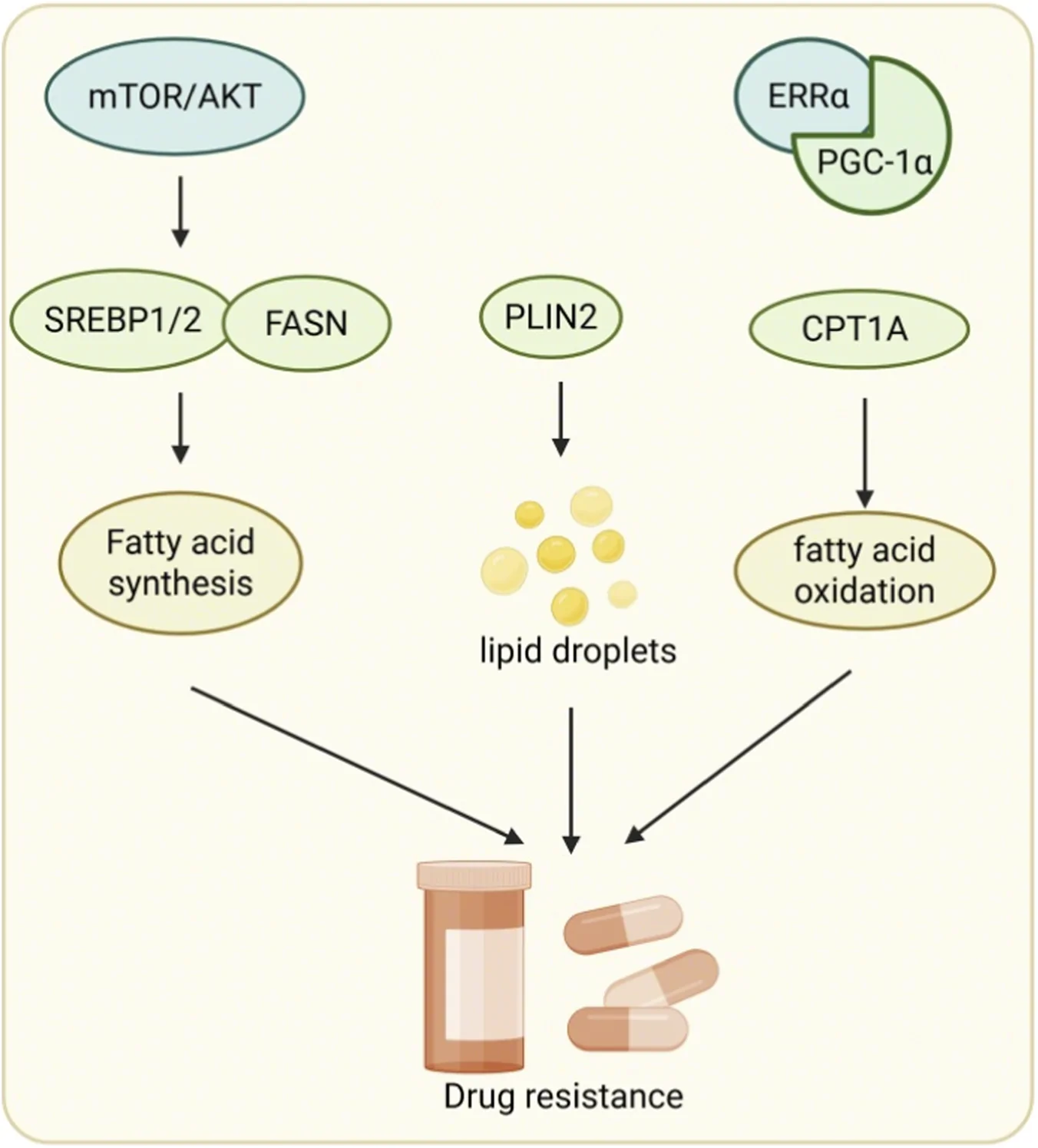
Lipid metabolic reprogramming and ferroptosis resistance in osteosarcoma. *De novo* lipogenesis (FASN, SREBP1/2) and fatty acid desaturation (SCD1) support membrane biosynthesis and protect against ferroptosis by reducing polyunsaturated fatty acid content. Fatty acid oxidation (CPT1A) provides an alternative energy source under metabolic stress. Lipid droplets (PLIN2) serve as lipid storage buffers. SCD1-mediated monounsaturated fatty acid production is a key determinant of ferroptosis sensitivity in osteosarcoma cells.

Lipid metabolic reprogramming has emerged as an equally critical driver of osteosarcoma progression and drug resistance, yet has been relatively understudied compared to glycolysis. Osteosarcoma cells exhibit enhanced *de novo* fatty acid synthesis, increased fatty acid uptake via CD36, dysregulated cholesterol metabolism, and altered lipid droplet dynamics. These alterations support membrane biogenesis for rapid proliferation, produce signaling molecules including phosphoinositides and eicosanoids, provide energy through fatty acid oxidation (FAO), and protect cells from lipid peroxidation and ferroptosis (Qiu et al., 2022) (Figure 3).

### 3.1 Fatty acid synthesis, desaturation, and ferroptosis resistance

Fatty acid synthase (FASN) is the multifunctional enzyme that catalyzes the *de novo* synthesis of palmitate from acetyl-CoA and malonyl-CoA. FASN is highly overexpressed in osteosarcoma compared to normal bone tissue, with expression levels correlating with tumor grade and poor prognosis. FASN inhibition using the small molecule inhibitor C75 reduces membrane lipid rafts, decreasing the activation of PI3K/AKT and ERK signaling pathways, and sensitizes osteosarcoma cells to cisplatin by reducing membrane fluidity and increasing drug penetration. Sterol regulatory element-binding proteins (SREBP1/2) are the master transcription factors regulating lipogenesis, activated downstream of mTORC1 signaling. SREBP1 directly drives the transcription of FASN, acetyl-CoA carboxylase (ACC), and stearoyl-CoA desaturase 1 (SCD1), while SREBP2 primarily regulates cholesterol synthesis genes including HMGCR (Schwab et al., 2024).

Stearoyl-CoA desaturase 1 (SCD1) catalyzes the rate-limiting step in the conversion of saturated fatty acids (SFAs) to monounsaturated fatty acids (MUFAs), and has recently been identified as a key mediator of ferroptosis resistance in osteosarcoma (Luo et al., 2023). By reducing the PUFA content of membrane phospholipids, SCD1 protects cells from lipid peroxidation and ferroptotic cell death. SCD1 inhibition sensitizes OS cells to ferroptosis inducers and chemotherapy. In multiple cancer types, ZEB1 — a master EMT transcription factor—has been shown to modulate SCD1 and other lipogenic enzymes, altering ferroptosis sensitivity (Nat Cell Biol, 2024). However, the specific ZEB1-SCD1-ferroptosis regulatory axis has not been directly validated in osteosarcoma models and represents a plausible but unconfirmed mechanism (Liu et al., 2022).

### 3.2 Fatty acid oxidation and lipid droplet dynamics

Carnitine palmitoyltransferase 1A (CPT1A) is the rate-limiting enzyme for mitochondrial fatty acid β-oxidation, conjugating long-chain fatty acids to carnitine for transport across the inner mitochondrial membrane. CPT1A is upregulated in chemotherapy-resistant osteosarcoma cells, where FAO serves as a critical alternative energy source when glycolysis is inhibited. FAO also maintains redox homeostasis by producing NADPH to support glutathione (GSH) reduction, and generates acetyl-CoA to feed into the TCA cycle and support histone acetylation. Estrogen-related receptor α (ERRα) directly transactivates CPT1A in cooperation with PGC-1α, and the CPT1A inhibitor etomoxir or ERRα inverse agonist XCT-790 significantly reduces FAO flux, increases ROS accumulation, and resensitizes resistant cells to chemotherapy (Rathore et al., 2021).

Lipid droplets (LDs) are dynamic organelles that store neutral lipids including triglycerides and cholesteryl esters, and play a critical role in protecting cells from lipotoxicity and oxidative stress. Perilipin 2 (PLIN2), the major coat protein of LDs, is highly expressed in drug-resistant osteosarcoma cells, where LDs sequester polyunsaturated fatty acids to prevent their peroxidation, buffer ER stress, and provide a rapidly mobilizable energy source during chemotherapy exposure. In cisplatin-resistant osteosarcoma cells, increased LD content correlates with resistance to ferroptosis and apoptosis, and inhibition of LD formation via PLIN2 knockdown or DGAT inhibitors sensitizes cells to chemotherapy. Additionally, LDs in tumor-associated macrophages transfer fatty acids to osteosarcoma cells via exosomes, further supporting FAO and drug resistance in the tumor microenvironment (Mohs et al., 2022).

### 3.3 Critical evaluation and open questions

Ferroptosis assay context. Most ferroptosis-resistance studies in OS use pharmacological inducers (erastin, RSL3) rather than chemotherapy drugs. Whether conventional chemotherapeutics induce ferroptosis as a primary cell death mechanism in OS remains debated. The clinical relevance of SCD1-mediated ferroptosis resistance for standard OS chemotherapy regimens therefore requires further validation.

Context-dependent SCD1 effects. Under lipid-replete conditions with serum, SCD1 inhibition may have limited effect because cells can scavenge MUFAs from the environment. This raises questions about *in vivo* efficacy, where lipid availability varies by tumor region.

Clinical translation barriers. SCD1 inhibitors that entered oncology trials failed primarily due to dose-limiting skin toxicities. FASN inhibitors also exhibit systemic toxicities related to impaired lipogenesis in normal tissues. The therapeutic window for lipogenic enzyme inhibition remains narrow.

## 4 Glutamine metabolic reprogramming and drug resistance in osteosarcoma

**FIGURE 4:**
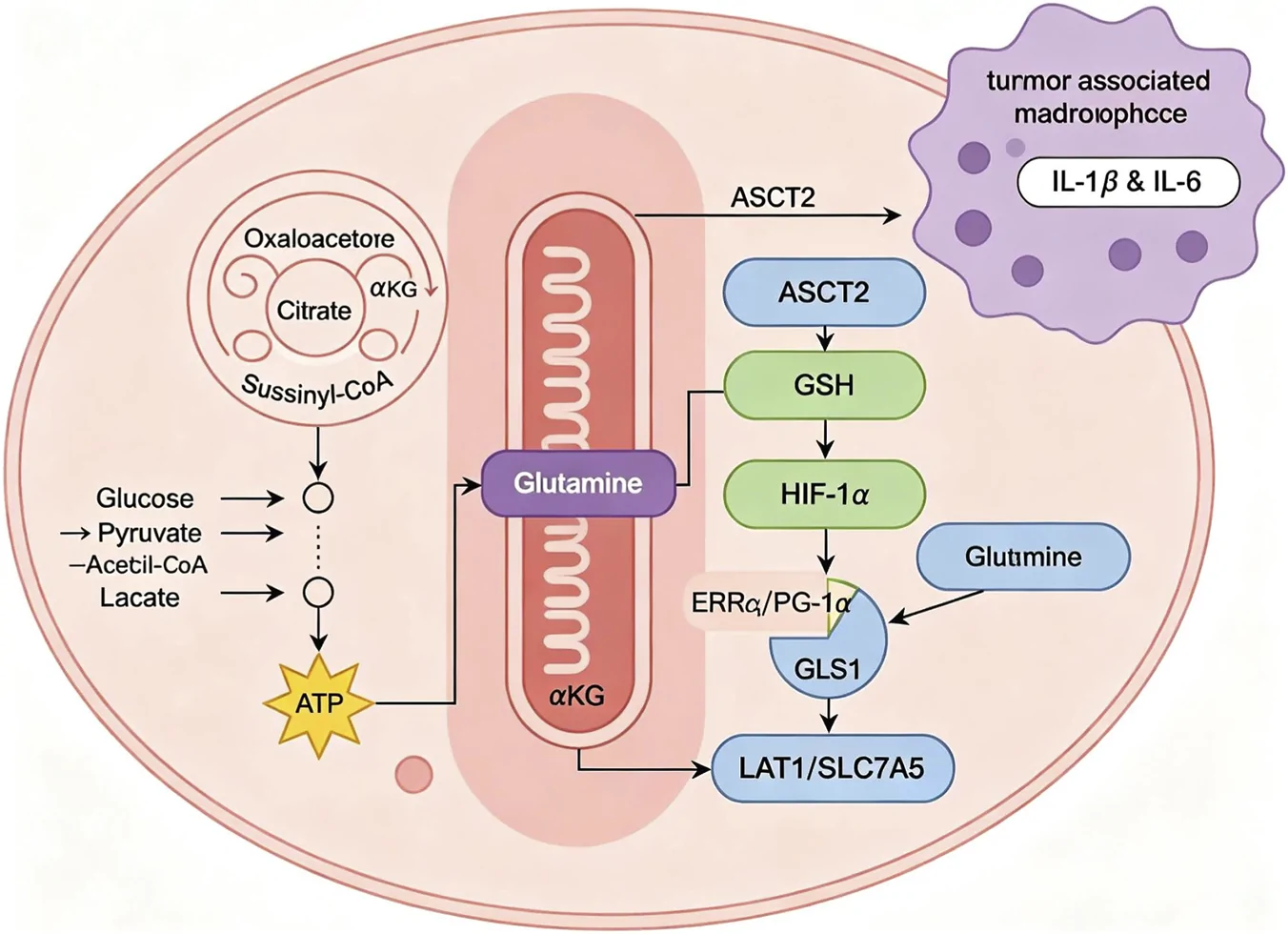
Glutamine metabolic reprogramming and its microenvironmental regulation in osteosarcoma. Glutamine enters cells through ASCT2 (SLC1A5) and LAT1/SLC7A5 transporters, and is catabolized by GLS1 to glutamate and α-KG, supporting TCA cycle anaplerosis and redox homeostasis (GSH synthesis). α-KG also serves as a cofactor for epigenetic regulators (histone demethylases, TET DNA demethylases). Tumor-associated macrophages secrete IL-1β and IL-6, further upregulating glutamine metabolism through HIF-1α and ERRα/PGC-1α signaling.

Glutamine is the most abundant amino acid in plasma and serves as a critical metabolic substrate for rapidly proliferating cancer cells, a phenomenon termed “glutamine addiction”. Osteosarcoma cells exhibit high rates of glutamine uptake and catabolism, which support energy production, macromolecular synthesis, redox homeostasis, and epigenetic regulation (Figure 4).

### 4.1 Glutamine uptake and catabolism

Glutamine enters osteosarcoma cells primarily through the neutral amino acid transporter ASCT2 (SLC1A5), with system L transporter LAT1 (SLC7A5) also contributing to glutamine uptake and mediating the efflux of glutamine in exchange for essential amino acids including leucine to activate mTORC1. ASCT2 is significantly upregulated in osteosarcoma patient samples and cell lines, with high expression correlating with advanced stage and poor survival. Pharmacological inhibition of ASCT2 using the competitive inhibitor V-9302 or benzylserine reduces glutamine uptake, inhibits mTORC1 signaling, and sensitizes osteosarcoma cells to chemotherapy (Teixeira et al., 2021).

Once inside the cell, glutamine is hydrolyzed by glutaminase (GLS1, kidney-type glutaminase) to glutamate and ammonia, in the first rate-limiting step of glutaminolysis. GLS1 is highly expressed in osteosarcoma, particularly in the more aggressive mesenchymal and telangiectatic subtypes, and its expression is directly induced by c-Myc and HIF-1α.Glutamate is subsequently converted to α-ketoglutarate (α-KG) by glutamate dehydrogenase (GLUD1) or alanine aminotransferase (ALT), which enters the TCA cycle to support oxidative phosphorylation and ATP production under conditions where glycolysis is suppressed. This anaplerotic function is critical for maintaining TCA cycle intermediates that are diverted into biosynthetic pathways. The selective, orally bioavailable GLS1 inhibitor telaglenastat (CB-839) has shown promising preclinical activity in osteosarcoma, reducing α-KG levels, inhibiting TCA cycle flux, and synergizing with chemotherapy and immunotherapy. Based on strong preclinical rationale, telaglenastat has been prioritized as one of the top novel agents for osteosarcoma clinical trials by the Children’s Oncology Group (Whittle et al., 2021; Jin et al., 2023).

### 4.2 α-KG-dependent epigenetic regulation and redox homeostasis

Beyond its metabolic roles, α-KG produced from glutaminolysis serves as an essential cofactor for α-KG-dependent dioxygenases, including Jumonji C domain-containing histone demethylases (JHDMs) and the TET family of DNA demethylases. Across multiple cancer types, α-KG-dependent H3K27me3 demethylation at ABC transporter gene promoters activates MDR1 and BCRP transcription, contributing to multidrug resistance. In osteosarcoma, glutamine addiction and high α-KG production are well-documented, and the epigenetic machinery for α-KG-dependent demethylation is intact. However, direct ChIP-grade evidence that α-KG-driven H3K27me3 demethylation specifically occurs at the ABCB1 and ABCG2 promoters in osteosarcoma cells is currently unavailable. This mechanism represents a strong hypothesis based on conserved epigenetic pathways but awaits direct experimental confirmation in OS models.

Glutamine metabolism is also critical for maintaining cellular redox homeostasis. Glutamate produced from glutaminolysis is the rate-limiting precursor for the synthesis of glutathione (GSH), the major cellular antioxidant that neutralizes ROS and maintains protein thiol groups in their reduced state. In chemotherapy-resistant osteosarcoma cells, upregulated glutaminolysis supports high GSH levels, which buffer chemotherapy-induced ROS and reduce oxidative damage to lipids, proteins, and DNA. Inhibition of GLS1 with CB-839 reduces GSH synthesis, increases ROS accumulation, and synergizes with cisplatin and doxorubicin by enhancing oxidative stress-induced cell death. Additionally, glutamine-derived aspartate and glycine provide precursors for nucleotide synthesis, supporting DNA repair and contributing to resistance against DNA-damaging chemotherapeutic agents.

### 4.3 Critical appraisal of glutaminolysis-targeting evidence

Variable response across models. Telaglenastat (CB-839) shows promising preclinical activity in some OS cell lines and xenografts, but response rates are highly variable. The molecular determinants of sensitivity—MYC amplification status, asparagine synthetase expression, compensatory pathway activity—remain incompletely characterized in osteosarcoma (Halama and Suhre, 2022).

Compensatory metabolic rewiring. Glutaminase inhibition triggers robust adaptive metabolic responses, including upregulated glucose oxidation. The mTORC1-dependent compensatory activation observed following PHGDH inhibition in osteosarcoma (Rathore et al., 2021) likely applies to other metabolic targets including GLS1, limiting single-agent durability.

Clinical trial experience from other cancers. Telaglenastat has shown modest single-agent activity across solid tumors. Common toxicities include gastrointestinal adverse effects (nausea, diarrhea, decreased appetite), fatigue, and reversible transaminitis. Osteosarcoma-specific trial results remain pending.

Bone microenvironment nutrient supply. The bone microenvironment is rich in glutamine supplied by osteoblasts, osteoclasts, and bone marrow stroma. This microenvironmental nutrient supply may reduce the efficacy of systemic glutaminase inhibition.

## 5 Metabolic pathway crosstalk and immunometabolic interactions in drug resistance

### 5.1 Crosstalk among glycolytic, lipid, and glutamine metabolic pathways

A critical limitation of previous metabolic research in osteosarcoma has been the study of individual metabolic pathways in isolation. In reality, glycolysis, lipid metabolism, and glutaminolysis are highly interconnected through shared signaling hubs, intermediate metabolite exchange, and reciprocal regulatory mechanisms, forming an integrated metabolic network that provides remarkable robustness and adaptability to tumor cells. This extensive crosstalk explains why single-agent metabolic inhibitors have shown limited clinical efficacy, as inhibition of one pathway is rapidly compensated by upregulation of alternative pathways.

At the signaling level, the PI3K/AKT/mTOR and HIF-1α/c-Myc axes function as master regulators that coordinately activate all three major metabolic pathways. mTORC1 simultaneously upregulates glycolysis by activating HK2 and PFKFB3, drives lipogenesis by activating SREBP1, and promotes glutaminolysis by upregulating c-Myc-dependent GLS1 expression. HIF-1α similarly induces GLUT1/LDHA for glycolysis, CD36 for fatty acid uptake, and GLS1 for glutaminolysis under hypoxic conditions. This coordinated regulation ensures that tumor cells have access to all necessary nutrients to support survival and proliferation under stress.

At the metabolite level, there is extensive exchange and recycling between pathways: glycolysis-derived dihydroxyacetone phosphate (DHAP) provides the glycerol backbone for triglyceride and phospholipid synthesis; glycolysis-derived pyruvate can be carboxylated to oxaloacetate to replenish TCA cycle intermediates (anaplerosis), complementing glutamine-derived α-KG; glutamine-derived citrate can be exported to the cytoplasm and converted to acetyl-CoA by ATP-citrate lyase (ACLY) to support *de novo* lipogenesis, providing an alternative carbon source for lipid synthesis when glucose is limited; and acetyl-CoA derived from fatty acid oxidation can inhibit glycolysis through allosteric regulation of pyruvate dehydrogenase (PDH), creating the “Randle cycle” metabolic switch. For example, when glycolysis is inhibited by 2-DG, osteosarcoma cells rapidly upregulate CPT1A and FAO to maintain ATP production, while simultaneously increasing GLS1 expression to support TCA cycle anaplerosis. This metabolic plasticity represents a major challenge for metabolic targeted therapy (Tan and Ning, 2024; Fritsche-Guenther et al., 2021).

### 5.2 Metabolic-immune crosstalk in the tumor microenvironment

Accumulating evidence demonstrates that metabolic reprogramming does not occur in tumor cells in isolation, but rather profoundly shapes the tumor immune microenvironment, and that immune cells in turn influence tumor cell metabolism. This bidirectional metabolic crosstalk is a critical, previously underappreciated driver of chemotherapy resistance and immunotherapy failure in osteosarcoma.

Lactate, the end product of aerobic glycolysis, is a key immunomodulatory metabolite. High lactate concentrations in the tumor microenvironment inhibit CD8^+^ cytotoxic T cell proliferation and cytokine production, suppress dendritic cell maturation, and promote polarization of macrophages toward the immunosuppressive M2 phenotype (Wang et al., 2021). M2 tumor-associated macrophages (TAMs) in turn secrete IL-6 and TGF-β that further activate glycolytic and lipogenic pathways in osteosarcoma cells, creating a feedforward immunosuppressive loop. Lactate also promotes the differentiation and recruitment of myeloid-derived suppressor cells (MDSCs), which inhibit T cell function through arginase and iNOS production. Furthermore, lactate inhibits the function of natural killer (NK) cells and reduces the efficacy of immune checkpoint inhibitors by upregulating PD-L1 expression on tumor cells and immune cells (Yu et al., 2022; Lu et al., 2022).

Lipid accumulation in the tumor microenvironment also exerts potent immunosuppressive effects. Lipid-laden tumor-associated macrophages exhibit impaired phagocytic activity and produce high levels of anti-inflammatory cytokines including IL-10 and TGF-β. Polyunsaturated fatty acids and cholesterol derivatives promote the differentiation of regulatory T cells (Tregs) while inhibiting effector T cell function. CD8^+^ T cells exposed to high lipid concentrations in the microenvironment take up excess fatty acids via CD36, leading to lipid peroxidation, mitochondrial dysfunction, and functional exhaustion—a state termed “lipotoxic T cell exhaustion”. Glutamine competition represents another important immunometabolic axis: tumor cells with high ASCT2 and GLS1 expression deplete glutamine from the microenvironment, limiting glutamine availability for T cells which require glutamine for activation, proliferation, and effector function (Wang et al., 2024). MDSCs also exploit glutamine metabolism to support their immunosuppressive functions, and GLS1 inhibition has been shown to reduce MDSC suppressive activity and enhance anti-tumor immunity (Liang et al., 2024; Hattinger et al., 2023).

## 6 Therapeutic strategies targeting metabolic reprogramming

### 6.1 Single-agent metabolic inhibitors: preclinical and clinical development

To date, no metabolic inhibitor has been approved for standard use in osteosarcoma, and clinical trial data in OS specifically remain very limited. Most evidence supporting metabolic targeting is derived from preclinical cell line and xenograft models, which incompletely predict clinical efficacy. Osteosarcoma’s rarity means dedicated trials are difficult to accrue, and most metabolic inhibitor studies include OS only as part of broad basket trials. Key metabolic targets and their clinical development status are summarized in Table 1.

**TABLE 1:** Key metabolic enzymes and therapeutic targets in osteosarcoma.

Enzyme	Pathway	Resistance mechanism	Evidence level in OS	Inhibitors	Clinical development status
HK2	Glycolysis	Glucose phosphorylation; anti-apoptotic signaling	✅ confirmed (OS cell lines, IHC)	2-DG, 3-BrPA, lonidamine	Phase 1/2 solid tumors; not OS-specific
PKM2	Glycolysis	Flux regulation; biosynthetic diversion; nuclear kinase hypothesized	✅ overexpression confirmed; ? nuclear H3T11 kinase unproven in OS	Shikonin, TEPP-46	Preclinical only
LDHA	Glycolysis	Lactate production; acidification; histone lactylation hypothesized	✅ overexpression confirmed; ? lactylation→MDR unproven in OS	FX11, gossypol	Preclinical
GLUT1	Glycolysis	Glucose uptake rate-limiting	✅ confirmed (OS tissue and cell lines)	WZB117, STF-31	Preclinical
SCD1	Lipid metabolism	MUFA synthesis; ferroptosis resistance	⚠ partial (ferroptosis shown; ZEB1 axis unvalidated in OS)	CAY10566, MF-438	Clinical candidates failed (skin toxicity)
FASN	Lipid metabolism	*De novo* lipogenesis; membrane biosynthesis	✅ confirmed for proliferation; resistance data limited	TVB-2640	Phase 1/2 solid tumors
CPT1A	Fatty acid oxidation	Alternative energy source under stress	⚠ partial (FAO upregulation in resistant subsets)	Etomoxir, perhexiline	Cardiac toxicity limits clinical use
GLS1	Glutaminolysis	Glutamine→glutamate; TCA anaplerosis; redox homeostasis	✅ confirmed (OS cell lines, PDX)	Telaglenastat (CB-839)	Phase 1/2; OS cohort accruing; modest single-agent activity
ASCT2/SLC1A5	Glutaminolysis	Glutamine import	⚠ partial (expression correlated with prognosis)	V-9302	Preclinical
LAT1/SLC7A5	Amino acid transport	Leucine/glutamine exchange; mTOR activation	⚠ partial (expression and prognostic data)	JPH203	Phase 1 solid tumors
PHGDH	Serine biosynthesis	Serine/glycine supply; nucleotides; redox balance	✅ OS-specific study (rathore et al., 2021)	NCT-503	Preclinical; mTORC1 compensation limits efficacy

Evidence level legend: ✅ Confirmed = direct experimental evidence in osteosarcoma models; ⚠ Partial = indirect or correlative evidence in OS; ❓ Hypothesized = demonstrated in other cancers but not yet validated in OS. Clinical status reflects current development phase, with most agents still in preclinical or early clinical testing and none yet approved for osteosarcoma standard care.

Lipid metabolism inhibitors include the FASN inhibitor TVB-2640 (denifanstat), which is currently in clinical trials for multiple solid tumors; the SCD1 inhibitor A939572; the CPT1A inhibitor etomoxir; and the ACC inhibitor firsocostat. Statin drugs (HMGCR inhibitors) which are widely used for cholesterol management have shown preclinical activity in osteosarcoma by inhibiting protein prenylation and disrupting lipid rafts, and retrospective studies suggest that statin use is associated with improved survival in osteosarcoma patients.

The most clinically advanced metabolic inhibitor for osteosarcoma is the GLS1 inhibitor telaglenastat (CB-839), formally prioritized by the Children’s Oncology Group New Agents for Osteosarcoma Task Force. However, clinical experience across other solid tumors shows that single-agent CB-839 has limited efficacy, with most responses observed in heavily pre-treated patients with specific molecular subtypes. Dose-limiting toxicities are primarily gastrointestinal: nausea, diarrhea, decreased appetite, and reversible transaminase elevation. Pharmacokinetic variability across patients is notable, and bone/bone marrow distribution of the drug has not been fully characterized. Osteosarcoma-specific trial results are eagerly awaited but not yet available; at present, the clinical benefit of telaglenastat in OS remains unproven (Lai et al., 2022).

### 6.2 Rational combination strategies: the future of metabolic therapy

Given the extensive metabolic crosstalk and plasticity discussed earlier, it is now widely recognized that single-agent metabolic therapy will unlikely be effective in osteosarcoma. Instead, rationally designed combination strategies that simultaneously target multiple metabolic pathways, or combine metabolic inhibitors with chemotherapy, immunotherapy, or targeted therapy, represent the most promising approach.

Dual metabolic pathway inhibition is one approach to overcome compensation. For example, combined inhibition of glycolysis (2-DG or FX11) and FAO (etomoxir) produces synergistic cytotoxicity in osteosarcoma cells by simultaneously blocking two major energy production pathways, with minimal toxicity to normal osteoblasts. Similarly, combined inhibition of GLS1 (CB-839) and FASN (TVB-2640) disrupts both energy production and membrane synthesis, leading to enhanced ER stress and apoptosis. The mTOR inhibitor rapamycin and its analogs (rapalogs) simultaneously inhibit glycolysis, lipogenesis, and glutaminolysis by targeting the master regulatory hub, explaining their preclinical activity in osteosarcoma (Park and Cheung, 2023; Wu et al., 2022).

Combination of metabolic inhibitors with immunotherapy to reverse immunosuppression is mechanistically attractive, but clinical data combining metabolic inhibitors with checkpoint inhibitors are limited and not specific to osteosarcoma. The immunologically “cold' nature of osteosarcoma—with relatively low mutational burden and T cell infiltration—may further limit benefit. Triple combination strategies incorporating a metabolic inhibitor, immunotherapy, and chemotherapy represent a mechanistically appealing but entirely hypothetical approach in osteosarcoma. No clinical trials have tested such a triple regimen. The complexity of triple-drug combinations—including overlapping toxicities, pharmacokinetic drug-drug interactions, and optimal sequencing—poses formidable translational challenges. Triple therapy should be regarded as a hypothesis-driven research direction, not a near-term clinical strategy.

### 6.3 Biomarker-driven precision metabolic therapy

Biomarker-guided patient selection will be critical for the successful clinical development of metabolic therapies in osteosarcoma. A “one-size-fits-all” approach is unlikely to succeed given the substantial metabolic heterogeneity (Table 2).

**TABLE 2:** Metabolic biomarkers for osteosarcoma.

Category	Specific biomarkers	Detection method	Clinical application	Validation status in OS
Metabolic imaging	^18^F-FDG (glucose uptake)	PET/CT	Staging; response monitoring	Clinical standard; non-specific; not predictive for metabolic drugs
Metabolic imaging	^18^F-FACBC (amino acid transport)	PET	Bone lesion characterization	Research/emerging; not validated for OS
Tissue protein biomarkers	HK2, PKM2, LDHA, GLUT1, SCD1, GLS1	Immunohistochemistry (IHC)	Prognostic stratification	Research-stage; no standardized scoring or clinical cutoffs
Circulating metabolites	Serum lactate; amino acid panels; lipid profiles	LC-MS, GC-MS, clinical chemistry	Prognosis; treatment monitoring	Research-stage; no clinically validated OS-specific panels
Liquid biopsy	Extracellular vesicle enzymes; cfRNA metabolic signatures	NGS, mass spectrometry	Non-invasive monitoring	Early research; not clinically available
Functional assays	Glycolytic rate; OXPHOS capacity; drug sensitivity	Seahorse flux analysis (PDO/PDX)	Predictive patient selection	Preclinical; not feasible for routine clinical use
Gene expression	Glycolysis signatures; glutaminolysis signatures; MYC signatures	RNA-seq, NanoString	Prognosis; patient stratification	Research-stage; no prospective validation in OS clinical trials

Biomarker categories span imaging, tissue protein, circulating metabolite, liquid biopsy, functional assay, and gene expression platforms. Most biomarkers remain at the research stage; ^18^F-FDG PET is the only metabolic biomarker currently in routine clinical use, primarily for staging rather than predictive guidance of metabolic therapy. Validated predictive biomarkers for patient selection remain a critical unmet need

#### 6.3.1 Imaging biomarkers

^18^F-FDG PET/CT is clinically available but non-specific and not validated as a predictive biomarker for glycolysis inhibitor response in OS. Novel tracers such as ^18^F-labeled glutamine analogs remain investigational (Li et al., 2024).

#### 6.3.2 Tissue-based protein biomarkers

IHC assessment of metabolic enzymes (HK2, PKM2, LDHA, SCD1, GLS1) is technically feasible but lacks standardized scoring algorithms and validated cutoffs for osteosarcoma. No metabolic IHC biomarker is currently used clinically to guide therapy selection in OS (Jimenez et al., 2022).

#### 6.3.3 Circulating metabolomic biomarkers

Serum metabolite profiling—including lactate, amino acid panels, and lipid signatures—offers a minimally invasive approach for patient stratification and treatment monitoring. Circulating extracellular vesicles carrying metabolic enzymes represent an emerging liquid biopsy strategy.

#### 6.3.4 Functional and genomic biomarkers

*Ex vivo* functional assays (Seahorse flux analysis on patient-derived organoids) can directly measure metabolic capacity and may predict drug sensitivity, but are not clinically feasible routinely. Metabolic gene expression signatures and MYC amplification status could potentially identify patients most likely to benefit, but prospective validation is lacking.

#### 6.3.5 Challenges in rare cancer biomarker development

Developing predictive biomarkers for a rare tumor like osteosarcoma is inherently challenging. Small patient populations limit statistical power, and multi-center collaboration is essential.

### 6.4 Barriers to clinical translation

Failed and disappointing clinical trials. The history of metabolic targeting in oncology includes many failures. Dichloroacetate showed only modest clinical activity despite strong preclinical rationale. Early-generation SCD1 inhibitors failed due to toxicity and lack of efficacy. Even telaglenastat has shown limited single-agent activity in most solid tumor types.

#### 6.4.1 Dose-limiting toxicities

Metabolic inhibitors target pathways essential for normal tissue function. GLS inhibitors cause GI toxicity; glycolysis inhibitors can cause hyperglycemia and fatigue; SCD1 inhibitors cause skin toxicities; FAO inhibitors can cause cardiac dysfunction.

#### 6.4.2 Pharmacokinetic and tissue distribution limitations

For osteosarcoma specifically, drug distribution into bone and the bone microenvironment is a critical but understudied question. Whether metabolic inhibitors achieve effective concentrations within bone tumors remains largely unknown.

#### 6.4.3 Tumor heterogeneity and adaptive resistance

Metabolic heterogeneity and plasticity are fundamental barriers to durable response. Tumors rapidly adapt through compensatory pathway switching, autophagy, and selection of resistant subclones.

#### 6.4.4 Regulatory and trial design challenges

Osteosarcoma’s rarity makes dedicated Phase 3 trials logistically and financially challenging. Basket trials and international collaboration are essential, but small OS subsets may be underpowered. Validated surrogate endpoints for OS metabolic trials are also lacking.

## 7 Challenges, limitations, and future directions

Despite significant progress in understanding the role of metabolic reprogramming in osteosarcoma drug resistance, several critical challenges remain that must be addressed to successfully translate these findings into clinical benefit for patients.

### 7.1 Key challenges and limitations

First and foremost is the remarkable metabolic plasticity and redundancy of osteosarcoma cells. Several distinct mechanisms contribute:

#### 7.1.1 Compensatory substrate switching

Glycolysis inhibition triggers compensatory glutaminolysis or FAO upregulation. Glutaminase inhibition leads to enhanced glucose oxidation. PHGDH inhibition in osteosarcoma activates mTORC1-dependent metabolic compensation that blunts therapeutic efficacy (Ottaviani and Jaffe, 2009).

#### 7.1.2 Mitochondrial adaptation and OXPHOS rescue

Many glycolysis-dependent tumors retain functional mitochondria that can be upregulated when glycolysis is inhibited. In osteosarcoma, emerging evidence suggests metastatic and cancer stem cell subpopulations are already more OXPHOS-dependent than bulk tumor cells.

#### 7.1.3 Autophagy

Autophagy is upregulated as a pro-survival adaptive response under metabolic inhibitor treatment, recycling damaged organelles and macromolecules. In osteosarcoma, autophagy is upregulated following chemotherapy and metabolic stress, and its inhibition (chloroquine, hydroxychloroquine) can sensitize OS cells to therapy.

#### 7.1.4 Macropinocytosis

Bulk uptake of extracellular proteins through macropinocytosis can supply amino acids when transporter-mediated uptake is insufficient. Well-characterized in KRAS-driven cancers, this mechanism is understudied in osteosarcoma and represents a plausible but unvalidated resistance mechanism.

#### 7.1.5 Redox system plasticity

Metabolic pathway inhibition disrupts redox homeostasis, triggering adaptive upregulation of Nrf2, glutathione, and thioredoxin antioxidant systems. This response can confer cross-resistance to ROS-inducing chemotherapies.

Second, systemic toxicity represents a major barrier to the clinical use of many metabolic inhibitors. Most metabolic pathways are essential for normal tissue function, particularly in rapidly proliferating tissues including the bone marrow, intestinal epithelium, and immune cells. For example, high-dose 2-DG causes significant neurotoxicity and hypoglycemia, while CPT1A inhibitors can cause cardiac toxicity due to the dependence of the heart on fatty acid oxidation. Developing tumor-specific delivery systems, such as antibody-drug conjugates or nanoparticle formulations that selectively deliver metabolic inhibitors to the tumor microenvironment, will be critical to widening the therapeutic window.

Third, the majority of preclinical studies have been performed using conventional two-dimensional cell culture models, which poorly recapitulate the complex three-dimensional bone microenvironment, nutrient gradients, hypoxia, and cellular heterogeneity present in patient tumors. Furthermore, most studies have focused on tumor cell intrinsic metabolism while neglecting the critical contributions of stromal cells, immune cells, and bone cells (osteoblasts, osteoclasts) in shaping the metabolic microenvironment. Metabolic symbiosis between different cell types in the bone microenvironment, such as the “reverse Warburg effect' where cancer-associated fibroblasts perform glycolysis and secrete lactate to feed tumor cell oxidative phosphorylation, remains poorly characterized in osteosarcoma (Casali et al., 2012; Xia et al., 2019).

### 7.2 Future directions and technological innovations

Technological advances will be critical to overcoming these challenges and advancing the field. Spatial metabolomics and mass spectrometry imaging (MSI) now allow the visualization of metabolite distributions within intact tumor sections at single-cell resolution, providing unprecedented insights into metabolic heterogeneity and cell-cell metabolic interactions in the tumor microenvironment. Single-cell RNA sequencing combined with metabolic flux analysis can define the metabolic states of individual tumor and immune cell populations, while CRISPR-Cas9 genetic screening can identify context-specific metabolic dependencies.

Patient-derived organoid (PDO) models that incorporate multiple cell types including immune cells and bone stromal cells will provide more physiologically relevant systems for testing metabolic therapies and identifying predictive biomarkers. Furthermore, the development of complex genetically engineered mouse models (GEMMs) of osteosarcoma that recapitulate the genomic heterogeneity and metastatic progression of human disease will be essential for evaluating the *in vivo* efficacy of metabolic combination therapies and assessing long-term toxicity.

Exciting emerging areas of research include the role of post-translational modifications such as histone lactylation, succinylation, and palmitoylation in linking metabolism to epigenetic regulation and drug resistance; the contribution of the gut microbiome to systemic metabolism and immunotherapy response in osteosarcoma; and the development of bifunctional molecules that simultaneously target metabolic enzymes and immune checkpoints. Additionally, exercise and dietary interventions including ketogenic diets and caloric restriction, which modulate systemic metabolism, may represent low-toxicity approaches to enhance the efficacy of metabolic inhibitors and immunotherapy.

## 8 Conclusion

Metabolic reprogramming represents a core hallmark of osteosarcoma that directly contributes to chemotherapy resistance through multiple integrated mechanisms involving energy production, biosynthesis, redox homeostasis, and epigenetic regulation. Glycolysis, lipid metabolism, and glutaminolysis form an interconnected network coordinated by mTOR, HIF-1α, and c-Myc signaling hubs. Metabolite-mediated epigenetic mechanisms—including histone lactylation and α-KG-dependent demethylation—represent emerging links between metabolism and gene expression that warrant further investigation in osteosarcoma.

### 8.1 Substantial challenges remain

Metabolic heterogeneity—across patients, between primary and metastatic sites, and within individual tumors—means no single metabolic target will be universally effective. Metabolic plasticity, through compensatory substrate switching, mitochondrial adaptation, autophagy, and redox defense, enables rapid emergence of resistance to single-agent inhibition. Most evidence remains preclinical, and no metabolic therapy has yet become standard of care for osteosarcoma.

Future progress will require: (1) rigorous clinical validation of lead compounds such as telaglenastat in well-designed OS-specific trials; (2) development of validated metabolic biomarkers for patient selection; (3) rational combination strategies that account for adaptive resistance mechanisms; and (4) advanced metabolomic technologies—including spatial and single-cell approaches—to better understand intratumoral metabolic heterogeneity. The biological rationale for metabolic targeting in osteosarcoma is strong, but successful clinical translation will require disciplined, biomarker-guided development rather than broad empiric application.
